# Effects of Smoking and Genotype on the PSR Index of Periodontal Disease in Adults Aged 18–49

**DOI:** 10.3390/ijerph9082839

**Published:** 2012-08-10

**Authors:** Deborah E. Polk, Xiaojing Wang, Eleanor Feingold, John R. Shaffer, Daniel E. Weeks, Robert J. Weyant, Richard J. Crout, Daniel W. McNeil, Mary L. Marazita

**Affiliations:** 1 Department of Dental Public Health and Information Management, University of Pittsburgh, School of Dental Medicine, Pittsburgh, PA 15261, USA; Email: rjw1@pitt.edu; 2 Department of Behavioral and Community Health Sciences, Graduate School of Public Health, University of Pittsburgh, Pittsburgh, PA 15261, USA; 3 Center for Craniofacial and Dental Genetics, School of Dental Medicine, University of Pittsburgh, Pittsburgh, PA 15219, USA; Email: xiw23@pitt.edu (X.W.); marazita@pitt.edu (M.L.M.); 4 Department of Oral Biology, School of Dental Medicine, University of Pittsburgh, Pittsburgh, PA 15261, USA; 5 Department of Human Genetics, Graduate School of Public Health, University of Pittsburgh, Pittsburgh, PA 15261, USA; Email: feingold@pitt.edu (E.F.); jrs51@pitt.edu (J.R.S.); weeks@pitt.edu (D.E.W.); 6 Department of Biostatistics, Graduate School of Public Health, University of Pittsburgh, Pittsburgh, PA 15261, USA; 7 Department of Periodontics, School of Dentistry, West Virginia University, Morgantown, WV 26506, USA; Email: rcrout@hsc.wvu.edu; 8 Departments of Psychology and Dental Practice and Rural Health, West Virginia University, Morgantown, WV 26506, USA; Email: dmcneil@wvu.edu; 9 Clinical and Translational Science Institute, and Department of Psychiatry, School of Medicine, University of Pittsburgh, Pittsburgh, PA 15213, USA

**Keywords:** adult, chronic periodontitis, genetics, genomics, smoking

## Abstract

Studies have found both genetic and environmental influences on chronic periodontitis. The purpose of this study was to examine the relationships among previously identified genetic variants, smoking status, and two periodontal disease-related phenotypes (PSR1 and PSR2) in 625 Caucasian adults (aged 18–49 years). The PSR Index was used to classify participants as affected or unaffected under the PSR1 and PSR2 phenotype definitions. Using logistic regression, we found that the form of the relationship varied by single nucleotide polymorphism (SNP): For rs10457525 and rs12630931, the effects of smoking and genotype on risk were additive; whereas for rs10457526 and rs733048, smoking was not independently associated with affected status once genotype was taken into consideration. In contrast, smoking moderated the relationships of rs3870371 and rs733048 with affected status such that former and never smokers with select genotypes were at increased genetic risk. Thus, for several groups, knowledge of genotype may refine the risk prediction over that which can be determined by knowledge of smoking status alone. Future studies should replicate these findings. These findings provide the foundation for the exploration of novel pathways by which periodontitis may occur.

## 1. Introduction

Chronic periodontitis is prevalent in the United States, with over one third of the dentate adult population having the disease [1]. People with chronic periodontitis can have problems chewing food and may ultimately experience tooth loss. Risk factors include the presence of oral pathogens that cause periodontal disease, variations to the host’s inflammatory response that may be genetically determined [2], and exposure to environmental factors such as smoking [3,4]. Among younger adults in particular, smoking appears to be associated with greatly increased risk of chronic periodontitis. In the National Health and Nutrition Examination Survey III, compared with nonsmokers, current smokers aged 20–49 years were 18.55 times as likely to have an average loss of attachment (*i.e.*, reduction in the connective tissue attaching the root of the tooth to the alveolar bone) of 3 mm or more (95% CI 9.44–36.45; [5]). For the 10% of younger adults with the greatest loss of attachment, the adjusted population attributable fraction due to current smoking was 60% [5]. Thus, smoking plays an important role in the etiology of periodontal disease in younger adults. 

Yet, as described above, smoking alone does not cause periodontal disease; pathogens and genetics also play roles. However, for the genotypes associated with chronic periodontitis, the relationship of those genotypes with smoking remains unknown at this time. There are three possible forms the relationship could take [6]. First, smoking and genes could contribute to separate causal pathways in chronic periodontitis (*i.e.*, additive effects). Second, smoking could up- or down-regulate the expression of genes that cause chronic periodontitis (*i.e.*, mediation); or genes could increase the probability of smoking, causing chronic periodontitis (*i.e.*, mediation). And third, smoking could cause chronic periodontitis in some people but not others, depending on their genes (moderation; see [7,8] for examples). Furthermore, the association between smoking and chronic periodontitis could differ depending on the genotype. Identifying the relationship between periodontally-related gene variants and smoking could help in assessing risk, targeting interventions to minimize disease risk, and improving the treatment of chronic periodontitis. 

In the present study using data from an observational study of younger adults, we assessed the association of smoking with a non-clinical indicator of periodontal health in the context of five locations in the genome where the allele tends to differ across people (*i.e.*, genotyped single nucleotide polymorphisms; SNPs) we previously identified in a genome-wide association scan (GWAS) of the same non-clinical indicator of periodontal health [9]. The phenotype is calculated from Periodontal Screening and Recording (PSR) index measures, which are non-clinical measures related to periodontal disease. The PSR index measures the depth of the periodontontal pocket from the free gingival margin to the bottom of the gingival sulcus. Clinical attachment level, the clinical measure of periodontal disease, measures the depth of the periodontal pocket from the cementoenamel junction to the bottom of the gingival sulcus. When the gingiva is not affected by either gingival inflammation or gingival recession, the free gingival margin and the cementoenamel junction will be the same, and the PSR index will provide an accurate measure of clinical attachment level. However, in cases where the gingiva is either inflamed or has receded, the free gingival margin and the cementoenamel junction will not be the same, and thus the PSR measure of the pocket will be inaccurate. In persons with gingival inflammation, it may overestimate disease, and in persons with severe gingival recession, it may underestimate disease [10]. Therefore, we view our phenotype as a measure of periodontal health and not a measure of chronic periodontitis.

## 2. Results and Discussion

### 2.1. Demographic and Descriptive Information about the Sample

The total sample size was 625 (63.8% female), with a mean age of 33.7 years (SD = 7.7, Min = 18, Max = 48.9). Using the PSR1 definition of disease, 94 participants were classified as affected (15.0%); whereas using the PSR2 definition of disease, 174 participants were classified as affected (27.8%). The two approaches to classifying disease resulted in different classifications for 80 participants (12.8% of the total sample). Using either PSR1 or PSR2, being affected was associated with greater age (Table 1).

**Table 1:** Results of logistic regression analyses relating age and smoking to PSR1 and PSR2.

		PSR1			PSR2		
Predictor	Level	OR	95% CI	Wald χ^2 a^	OR	95% CI	Wald χ^2 a^
Age	10 year increments	1.35	1.01–1.81	4.04 *	1.59	1.25–2.01	14.57 ***	
Smoking	Never	1.00		3.29	1.00		13.29 **	
	Former	1.07	0.60–1.92		1.15	0.72–1.84		
	Current	1.57	0.92–2.70		2.13	1.36–3.31		

Note: Age was included as a covariate in the smoking model. PSR1 = missingness in completely edentulous sextants is attributed to causes other than periodontal disease. PSR2 = missingness in completely edentulous sextants is attributed to periodontal disease; * *p* < 0.05; ** *p* < 0.001; *** *p* < 0.0001; ^a^ Degrees of freedom = 1 for age and 2 for smoking.

**Figure 1:**
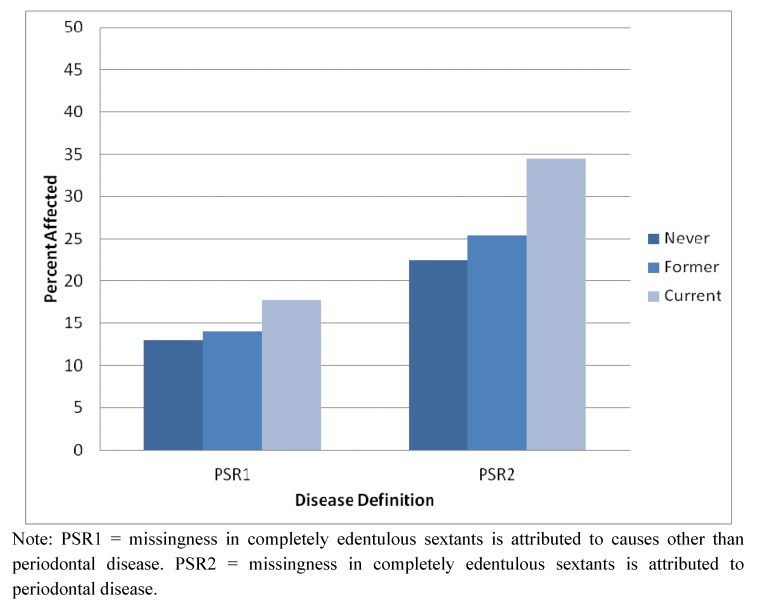
Relationship of smoking status with PSR1 and PSR2.

With respect to smoking, 200 (32.0%) participants never smoked; 193 (30.9%) participants were former smokers; and 232 (37.1%) participants were current smokers. Among current smokers, the average number of cigarettes smoked per week was 94.9 (SD = 59.9). Table 1 and Figure 1 present the relationship between smoking status and disease status. As expected, relative to never smokers, current smokers had higher odds of having disease (Table 1). Odds of having disease did not differ between former smokers and never smokers; therefore, for analyses below, we collapse former and never smokers into one category.

### 2.2. Results Supporting an Additive Relationship

In our previous study [9], we found an association between PSR1 and rs10457525. When PSR1 was predicted by age, smoking status, and rs10457525 genotype simultaneously, age in 10-year increments (OR = 1.46, 95% CI = 1.07 to 1.98), smoking status (OR = 1.62, 95% CI = 1.02 to 2.59), and genotype (GG *vs.* TT: OR = 0.17, 95% CI = 0.07 to 0.40; TG *vs.* TT: OR = 0.34, 95% CI = 0.14 to 0.82) each accounted for unique variance in the outcome. 

Similarly, in our previous study, we found an association between PSR2 and rs12630931. When PSR2 was predicted by age, smoking status, and rs12630931 genotype simultaneously, age in 10-year increments (OR = 1.71, 95% CI = 1.33 to 2.20), smoking status (OR = 2.00, 95% CI = 1.37 to 2.93), and genotype (CC *vs.* TT: OR = 3.54, 95% CI = 1.87 to 6.69; CT *vs.* TT: OR = 1.86, 95% CI = 1.26 to 2.74) each accounted for unique variance in the outcome. 

For neither rs10457525 nor rs12630931 was the effect of genotype moderated by an interaction with smoking status. Using Chi-square analysis, neither SNP was related to smoking status. Thus, genotype does not mediate the association of affected status with smoking status; and smoking status does not mediate the association of affected status with genotype. Thus, for both rs10457525 and rs12630931, an additive model with smoking best described the relationship with PSR1 or PSR2. To the best of our knowledge, none of the genes near these SNPs produces a protein known to be related to periodontal disease or its two major risk factors, smoking and diabetes.

### 2.3. Results Supporting a Unique Relationship for Genotype but Not Smoking Status

In our previous study, we found an association between PSR1 and rs10457526. When PSR1 was predicted by age, smoking status, and rs10457526 genotype simultaneously, age in 10-year increments (OR = 1.47, 95% CI = 1.08 to 2.00) and genotype (GG *vs.* TT: OR = 0.14, 95% CI = 0.06 to 0.32; TG *vs.* TT: OR = 0.26, 95% CI = 0.11 to 0.59) each accounted for unique variance in the outcome, and smoking status marginally accounted for variance (OR = 1.55, 95% CI = 0.97 to 2.47). 

Similarly, in our previous study, we found an association between PSR1 and rs733048. When PSR1 was predicted by age, smoking status, and rs733048 genotype simultaneously, age in 10-year increments (OR = 1.42, 95% CI = 1.05 to 1.93) and genotype (AA *vs.* GG: OR = 4.46, 95% CI = 1.93 to 10.29; AG *vs.* GG: OR = 2.23, 95% CI = 1.40 to 3.57) each accounted for unique variance in the outcome, and smoking status was not associated. 

For neither rs10457526 nor rs733048 was the effect of genotype moderated by an interaction with smoking status. Using Chi-square analysis, neither SNP was related to smoking status. Thus, genotype does not mediate the association of affected status with smoking status; and smoking status does not mediate the association of affected status with genotype. Thus, for rs10457526 and rs733048 using the PSR1 definition, smoking was no longer associated with the outcome once genotype was taken into consideration. To the best of our knowledge, none of the genes near these SNPs produces a protein known to be related to periodontal disease or its two major risk factors, smoking and diabetes. 

### 2.4. Results Supporting an Interaction of Genotype by Smoking Status

**Figure 2:**
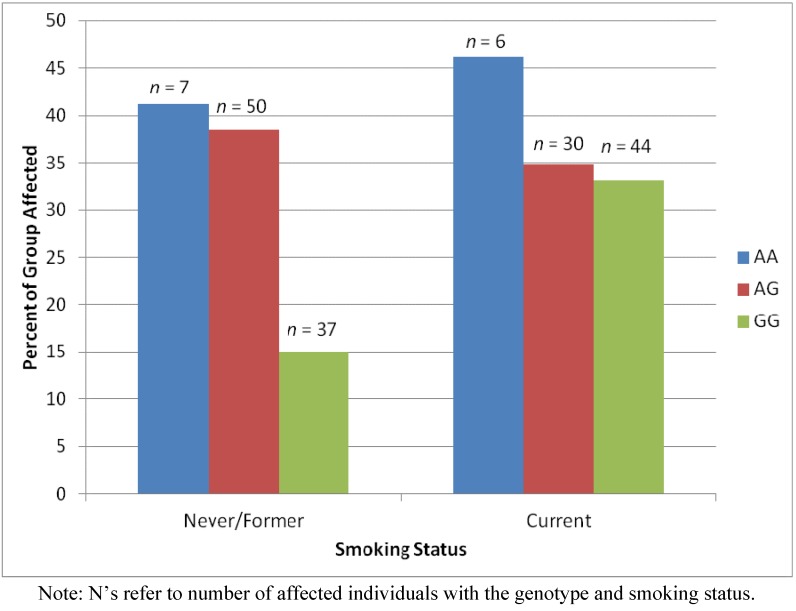
Percents of persons within groups defined by both smoking status and rs733048 genotype who are affected (PSR2).

In our previous study, we also found an association between PSR2 and rs733048. When PSR2 was predicted by age, smoking status, and rs733048 genotype simultaneously, they each accounted for unique variance in the outcome. However, the main effect of the rs733048 genotype on affected status was moderated by an interaction with smoking status (Wald χ^2^ (df = 2) = 10.05, *p* < 0.007; Figure 2). Among those with the GG genotype, as expected, never and former smokers were less likely than current smokers to be cases (OR = 0.30, 95% CI = 0.18 to 0.50). However, among those with either the AA or the AG genotypes, current smoking was not associated with an increased or decreased risk of being a case relative to never smoking or formerly smoking. To determine whether having the AA or AG genotypes protected current smokers or increased the risk of former and never smokers, we examined the interaction the other way. Relative to having the GG genotype, having the AA genotype (OR = 3.73, 95% CI = 1.30 to 10.74) or the AG genotype (OR = 3.60, 95% CI = 2.17 to 5.97) was associated with an increased risk among never and former smokers. Relative to having the GG genotype, having the AA (OR = 2.22, 95% CI = 0.69 to 7.13) or AG genotype (OR = 1.03, 95% CI = 0.57 to 1.85) was not associated with increased risk among current smokers. Thus, having either the AA or AG genotypes was associated with an increased risk of having disease among former and never smokers.

**Figure 3:**
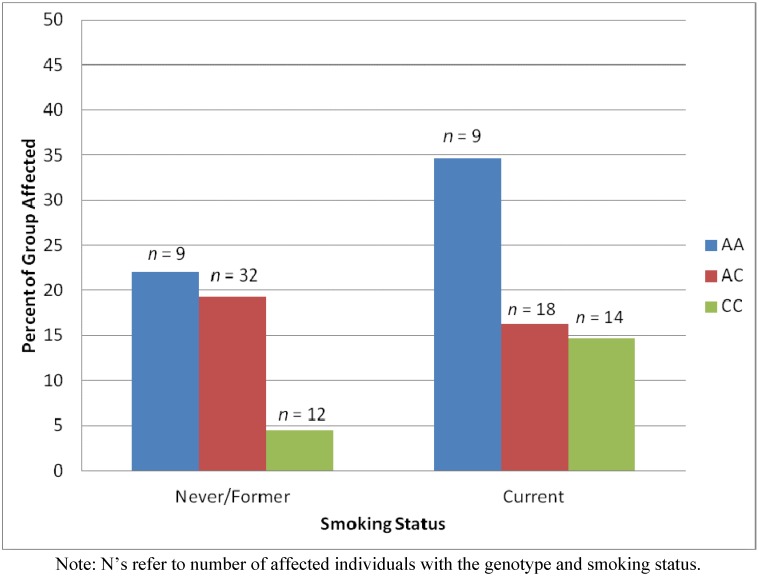
Percents of persons within groups defined by both smoking status and rs3870371 genotype who are affected (PSR1).

In our previous study, we found an association between PSR1 and rs3870371. When PSR1was predicted by age, smoking status, and rs3870371 genotype simultaneously, age and genotype accounted for unique variance in the outcome and smoking status was not associated. However, the main effect of the rs3870371genotype on case status was moderated by an interaction with smoking status (Wald χ^2^ (df = 2) = 5.65, *p* < 0.06; Figure 3). Among those with the CC genotype, as expected, never and former smokers were less likely than current smokers to be cases (OR = 0.34, 95% CI = 0.15 to 0.77). However, among those with either the AA or the AC genotypes, current smoking was not associated with an increased or decreased risk relative to never smoking or formerly smoking. To determine whether having the AA or AC genotypes protected current smokers or increased the risk of former and never smokers, we examined the interaction the other way. Relative to having the CC genotype, having the AA genotype (OR = 4.35, 95% CI = 1.68 to 11.25) or the AC genotype (OR = 3.74, 95% CI = 1.85 to 7.58) was associated with an increased risk among never and former smokers. Relative to having the CC genotype, having the AA genotype (OR = 2.85, 95% CI = 1.05 to 7.73) was associated with an increased risk among current smokers. Relative to having the CC genotype, having the AC genotype was not associated with an increased or decreased risk among current smokers. Thus, having either the AA or AC genotypes was associated with an increased risk among former and never smokers and having the AA genotype was associated with an increased risk among current smokers. 

Using Chi-square analysis, neither SNP was related to smoking status. Thus, genotype does not mediate the association of affected status with smoking status; and smoking status does not mediate the association of affected status with genotype.

Thus, the relationship of rs733048 with PSR2 was moderated by smoking. Among people with the GG genotype, the relationship between smoking and risk was as expected: current smoking was associated with higher risk. However, having either the AA or AG genotypes was associated with an increased risk among former and never smokers. This identifies two new groups of people at higher risk. Similarly, the relationship of rs3870371 with PSR1 was moderated by smoking. Among people with the CC genotype, the relationship between smoking and risk was as expected: current smoking was associated with higher risk. However, having either the AA or AC genotypes was associated with an increased risk among former and never smokers and having the AA genotype was associated with an increased risk among current smokers. This identifies three new groups of people at higher risk. Although none of the genes near rs733048 is known to be related to periodontal disease or its two major risk factors, smoking and diabetes, there are two genes, HAS2 and HAS2AS, near rs3870371 that have previously-documented relationships with the periodontium. They are both related to wound healing [11,12].

## 3. Experimental Section

### 3.1. Participant Recruitment

As previously described [13], the study population was drawn from four, economically distressed, rural counties in northern West Virginia and western Pennsylvania in which the Center for Oral Health Research in Appalachia is active. Families were eligible if they had at least one adult and at least one child between the ages of 1 and 18 biologically-related to that adult who lived together; recruitment was not based on oral health status. Using these criteria, we were able to recruit 650 families. Once a family was recruited, everyone living in the household was invited to participate, regardless of biological or legal relationship. Written informed consent was obtained from all adult participants. All study procedures and consent forms were approved by the Institutional Review Boards of the University of Pittsburgh and West Virginia University. 

### 3.2. Phenotype and Covariate Assessment

Participants received a comprehensive orodental examination by a licensed dentist or dental hygienist in a well equipped, modern dental operatory. Data for each study participant included periodontal status and history, medical history, health behaviors, social adjustment, demographics (ethnicity, SES, *etc*.), and DNA source (blood, saliva). As previously described [9], to assess periodontal status, a modified Periodontal Screening and Recording (PSR) procedure [14] was performed as follows. Except for third molars, which were excluded, every tooth was evaluated. The mouth was divided into sextants, and the probing depth for the deepest pocket in each sextant was recorded. If all teeth in a given sextant were missing (*i.e.*, the sextant was completely edentulous), no observation was recorded for that sextant. Because the reasons why the teeth were missing were unknown, we created two definitions of disease. In the PSR1 definition of disease, teeth in edentulous sextants were treated as though they had not had periodontal disease (*i.e.*, pocket probing depth less than 5.5 mm). In the PSR2 definition of disease, teeth in edentulous sextants were treated as though they had had disease (*i.e.*, pocket probing depth of at least 5.5 mm). Affected persons were defined as persons with at least two sextants with a pocket probing depth of at least 5.5 mm or self-reported “gum surgery” (n = 14). Persons not classified as affected were classified as unaffected. 

To assess smoking, we asked the following questions “Please indicate which drugs you have ever tried for recreation by circling yes. If you have never tried the drug, skip to the next drug on the list. DR02–smoking tobacco (Cigarettes, Pipes, Cigars)” from the Drug Use Screening Inventory-Revised (DUSI-R; [15]) and “Are you currently a daily cigarette smoker?”. From these two questions, we were able to distinguish never, former, and current smokers. We compared urinary and salivary cotinine levels as assayed by NicAlert strips to self-reported smoking status in 59 adults with no incentive to lie. There was 90% and 86% agreement between self-report and urinary or salivary cotinine, respectively. 

Genotyping, imputation, and quality control have been previously described [9]. To reduce Type I error due to population stratification, the sample on which the GWAS was conducted was limited to self-reported non-Hispanic Caucasians only. 

### 3.3. Data Analysis

To minimize the risk of an inflated type I error due to misclassifying non-diseased persons as having disease, we excluded from our analyses persons who were pregnant (*n* = 13) or who reported taking medications that could result in gingival hyperplasia or edema including birth control pills (*n* = 24), estrogen replacement therapy (*n* = 3), calcium channel blockers (*n* = 1), or Dilantin (*n* = 4). GWAS analysis identified 11 SNPs having suggestive associations with either PSR1 or PSR2 (9). In the present study, we examined only those five SNPs that were genotyped and not those that were imputed (see Table 2).

**Table 2:** Genotyped SNPs identified by GWAS.

SNP	Outcome Associated With	Strength of Association	Chromosome, Coordinate	Nearby Genes
rs10457525	PSR1	5.72 × 10^−07^	6, 129872966	LAMA2, ARHGAP18 (SENEX)
rs733048	PSR1	1.07 × 10^−06^	4, 13242797	HSP90AB2P, RAB28, BOD1L, NKX3-2
rs10457526	PSR1	1.17 × 10^−06^	6, 129896501	LAMA2, ARHGAP18 (SENEX)
rs733048	PSR2	6.15 × 10^−06^	4, 13242797	HSP90AB2P, RAB28, BOD1L, NKX3-2
rs12630931	PSR2	7.32 × 10^−06^	3, 31981767	OSBPL10, ZNF860, GPD1L, CMTM8, STT3B
rs3870371	PSR1	8.79 × 10^−06^	8, 122697132	HAS2, HAS2AS

To examine additive effects of smoking status (current *versus* never and former) and each of the selected SNPs, we included both smoking status and SNP genotype in logistic regression models simultaneously, covarying age. Predictors were considered to account for unique variance in affected status (*i.e.*, additive effects) if they were associated with being affected at *p* < 0.05. To test for mediation, we first examined whether smoking status (current *versus* never and former) was associated with each of the selected SNPs. Using Chi-square analysis, none of the SNPs was related to smoking status. Thus, we were able to rule out both forms of mediation. To examine the ability of smoking status (current *versus* never and former) to moderate the relationship between genotype (AA, Aa, and aa) and affected status (affected *versus* unaffected), we used logistic regression covarying age and main effects. In our power calculations for PSR1, we could achieve 15–80% power for interactions by making assumptions for parameters based on what we observed in our study (e.g., sample size, allele frequency, disease prevalence, and effect sizes for the SNPs and smoking). For PSR2, we could achieve 29–96% power for interactions. Because in the GWAS [9], four of the five SNPs were associated with either PSR1 or PSR2 but not both, for those SNPs, we examined pathways with only the disease definition for which the association had been observed. Because this was an exploratory study, no correction was made for multiple comparisons. 

### 3.4. Missing Data

Genotyping was available for 694 participants. Due to missing values on smoking status, 69 participants were excluded from analyses involving smoking status.

## 4. Conclusions

Smoking is a known risk factor for chronic periodontitis [3] and as expected, in our sample, current smoking was associated with having at least two sextants with periodontal probing depths exceeding 5.5 mm. Furthermore, we determined the relationship between genotypes previously identified as being associated with periodontal probing depths exceeding 5.5 mm in this sample and smoking. For rs10457525 under the PSR1 definition of affected status and rs12630931 under the PSR2 definition of affected status, we demonstrated that the effects of smoking and genotype on risk were additive. For rs10457526 and rs733048 under the PSR1 of affected status, smoking was no longer associated with having periodontal probing depths exceeding 5.5 mm once genotype was taken into consideration. Finally, we found that smoking moderated the relationships of rs3870371 under the PSR1 definition and rs733048 under the PSR2 definition with affected status. Thus, for several groups of people, knowledge of genotype may refine the risk prediction over that which can be determined by knowledge of smoking status alone. This is consistent with other studies that have also obtained evidence that smoking moderates the relationship between genotype and periodontal disease [3]. 

### Strengths and Limitations

This study has several strengths and limitations. One strength of the study is that we examined three possible relationships genotype and smoking could have with affected status. The study had some limitations as well. First, the phenotype was defined based on measurement of the PSR index, which is a non-clinical measure related to periodontal disease. In addition, there could be misclassification error due to missing teeth, although we attempted to address this issue by using two definitions. Future studies should improve the definition of the phenotype. Finally, we may have had low power to detect interactions. Future studies should include more affected persons.

In this study, we determined the relationships between SNPs, smoking status, and affected status. By doing this, we identified several groups that were at higher risk than would have been predicted from their smoking status alone. These findings are significant, because they provide the foundation for more precise risk assessment and the identification of new preventive and treatment interventions. Future studies should confirm the pathways we observed in the present study. 
